# Raise quantum anomalous Hall states up

**DOI:** 10.1093/nsr/nwaa214

**Published:** 2020-04-02

**Authors:** Ke He

**Affiliations:** State Key Laboratory of Low-Dimensional Quantum Physics, Department of Physics, Tsinghua University, China; Frontier Science Center for Quantum Information, China; Beijing Institute of Quantum Information Science, China

Experimental progress on the quantum anomalous Hall (QAH) effect has been significantly accelerated recently by the discovery of an intrinsic magnetic topological insulator MnBi_2_Te_4_ [1]. The material is natively antiferromagnetic, but an external magnetic field of several tesla can overcome its weak interlayer antiferromagnetic coupling, making it ferromagnetic. Interestingly, ferromagnetic MnBi_2_Te_4_ is predicted to be a magnetic Weyl semimetal, a topological phase hunted for almost a decade but with few cases confirmed experimentally [2]. A characteristic property of a magnetic Weyl semimetal is that its thin films can show the QAH effect with the Chern number (*C*), i.e. the number of the dissipationless edge channels, increasing with their thicknesses [3]. It provides an elegant way to engineer the QAH edge states for various studies and applications, but has never been experimentally demonstrated.

**Figure 1.**
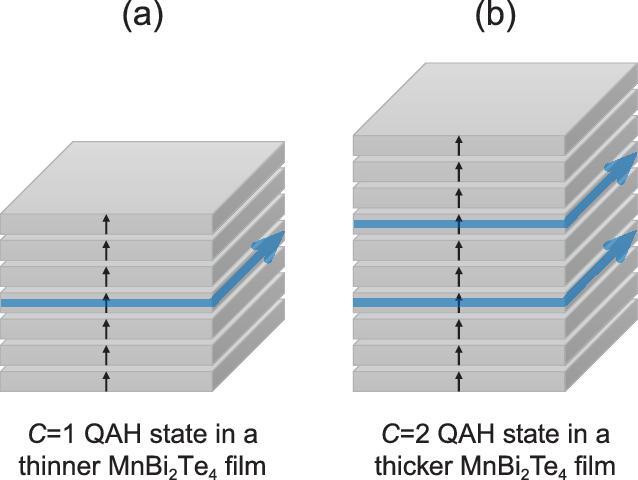
Schematics of the *C* = 1 QAH state (a) and the *C* = 2 QAH state (b) in thinner (7 septuple-layer) and thicker (10 septuple-layer) MnBi_2_Te_4_ films, respectively. The black arrows indicate the magnetization vectors. The blue lines with arrows indicate the chiral edge states.

In a recent work published in *National Science Review*, Prof. Jian Wang from Peking University and his collaborators observed the Hall resistance plateaus of both one quantum resistance (∼25.8 kΩ) and half quantum resistance (∼12.9 kΩ), corresponding to the *C *= 1 and *C *= 2 QAH states, respectively, in MnBi_2_Te_4_ flakes of different thicknesses under a moderate magnetic field of about 5 tesla (Fig. 1) [4]. This unambiguously confirms the magnetic Weyl semimetal phase in ferromagnetic MnBi_2_Te_4_, and, for the first time, showed us the unique aspect of magnetic Weyl semimetals.

An astonishing observation is that the QAH states can survive rather a high temperature in MnBi_2_Te_4_ flakes. *C *= 2 QAH state is observed at T >13 K. In some *C *= 1 samples, almost quantized anomalous Hall resistance is observed at a temperature even higher than the magnetic ordering temperature (90.4% at 45 K in a seven-septuple-layer device, 96.7% at 30 K in an eight-septuple-layer device). This appears counter-intuitive, but is actually a natural result of the two-dimensional magnetism of MnBi_2_Te_4_. According to the Mermin-Wagner theorem, the ordering temperature of such a 2D magnetic system is not limited by the exchange energy but the magnetic anisotropic energy, which suppresses the magnetic fluctuation resulting from low dimension. A perpendicular magnetic field increases the effective anisotropic energy and thus elevates the effective magnetic ordering temperature. The topological electronic states of MnBi_2_Te_4_ are predicted to have a large magnetically induced gap (several tens of meV), which can, in principle, support the QAH state above room temperature if the magnetic ordering temperature could also reach so high. The present work strongly supports such a robust QAH state in it.

The additional magnetic anisotropy is not necessarily provided by an external magnetic field. Exchange coupling with a neighboring ferromagnetic or antiferromagnetic insulator can also stabilize the long-range magnetic order of MnBi_2_Te_4_. A recent theoretical work showed that in some magnetic van der Waals materials, the strength of interlayer magnetic coupling exceeds 10 meV, which implies the possibility of elevating the magnetic ordering temperature of MnBi_2_Te_4_ above 77 K by choosing appropriate neighboring layers, without the need for an external magnetic field [5]. The QAH effect and its electronic applications above liquid nitrogen temperature may be achieved in such MnBi_2_Te_4_-based heterostructures.

***Conflict of interest statement.*** None declared.
